# Long-term follow-up of “three horse shoe-like incision (3-HSI) holmium laser enucleation of the prostate: outcomes and efficacy of a novel en-bloc technique for anatomic transurethral prostatectomy”

**DOI:** 10.1186/s12894-025-01885-6

**Published:** 2025-07-31

**Authors:** Maximilian Glienke, MF von Bargen, A Özkan, D Sirtl, C Gratzke, A Miernik, D Schoeb

**Affiliations:** Department of Urology, Faculty of Medicine, Medical Centre, University of Freiburg, Hugstetter Str. 55, 79106 Freiburg, Germany

**Keywords:** Benign prostatic obstruction, BPO, Laser therapy/Methods, Lower urinary tract symptoms, LUTS, Prostatic hyperplasia/Surgery

## Abstract

**Purpose:**

This study aims to evaluate the long-term outcomes of the novel “three horseshoe-like incision” (3-HSI) technique for holmium laser enucleation of the prostate (HoLEP) compared to the traditional three-lobe HoLEP method in treating benign prostatic hyperplasia (BPH).

**Methods:**

A comparative study was conducted of 54 patients undergoing the 3-HSI technique and 27 patients treated with the three-lobe technique from November 2016 to June 2018. All surgeries were performed by a single experienced surgeon. Data were collected preoperatively, perioperatively, and during follow-up. Statistical analyses were conducted using the Wilcoxon rank-sum test and Fisher’s exact test, with significance set at *p* < 0.05.

**Results:**

Preoperative parameters showed no significant differences between groups, except for a higher symptom burden and medication use in the 3-HSI group. The 3-HSI technique revealed a significantly shorter perioperative operative duration (44.9 ± 43 vs. 60.1 ± 34 min, *p* = 0.01). Hospital stay, catheterization period, and resected tissue weight were similar. Follow-up data revealed similar functional outcomes and patient satisfaction between groups, with no significant differences in complication rates.

**Conclusion:**

The 3-HSI HoLEP technique demonstrates similar safety and efficacy to the traditional method, with the added benefit of shorter operative time. These findings support the 3-HSI technique as a viable and potentially superior option for BPH surgical intervention.

**Trial registration:**

This trial has been registered in the German Clinical Trials Register DRKS-ID: FRKS005540.

**Supplementary Information:**

The online version contains supplementary material available at 10.1186/s12894-025-01885-6.

## Introduction

Benign prostatic hyperplasia (BPH) is a common condition in aging men, often leading to lower urinary tract symptoms (LUTS) that significantly impact quality of life [1]. While transurethral resection of the prostate (TURP) has been the historical gold standard for surgical management, holmium laser enucleation of the prostate (HoLEP) has emerged as a superior alternative, particularly for larger prostates, due to its lower bleeding risk, shorter catheterization time, and long-term efficacy [2, 3].

One of the key refinements in HoLEP techniques has been the development of en-bloc enucleation methods, which aim to streamline the procedure by removing the prostate adenoma in a more anatomically controlled fashion. Several en-bloc approaches have been introduced, each with distinct modifications to improve operative efficiency and functional outcomes [4, 5].

The Three Horse-Shoe Incision (3-HSI) technique represents a novel variation of en-bloc HoLEP, designed to enhance surgical control and efficiency by utilizing three strategically placed incisions to facilitate enucleation. In contrast to the traditional three-lobe HoLEP technique, where the adenoma is divided into separate lobes before removal, the 3-HSI method maintains tissue integrity throughout the procedure. One hypothesized advantage of en-bloc techniques, including 3-HSI, is the potential reduction of postoperative stress urinary incontinence by preserving anatomical structures [6].

In this study we are assessing the long-term outcomes of the “three horseshoe-like incision” HoLEP technique. Our research builds upon our initial findings described in the original article entitled “Three horse shoe‐like incision” holmium laser enucleation of the prostate: first experience with a novel en bloc technique for anatomic transurethral prostatectomy [7]. Through a comprehensive follow-up, we seek to determine the effectiveness, safety, and durability of this novel technique. By providing detailed long-term data, we hope to contribute to the ongoing evolution of surgical approaches for BPH and validate the “three horse shoe‐like incision” HoLEP as a viable and superior option for our patients.

## Materials and methods

This study involved a comparison between the 3-HSI technique [7] and classical three-lobe technique by Gilling et al. [8]. We included 54 patients who underwent 3-HSI between November 2016 and June 2018 at the Department of Urology, University of Freiburg Medical Center, Germany. They were compared to 27 patients from our prospectively collected HoLEP database, who underwent the three-lobe technique. All surgeries were performed by a single experienced surgeon (AM) to ensure a consistent operative technique. Patients underwent laser enucleation for symptomatic BPH in cases of unsatisfactory results with medical therapy, unsuccessful catheter removal after urinary retention, or progressive increase in postvoid residual volume.

3-HSI utilized a 26-Fr continuous-flow laser resectoscope equipped with 12° optics (SHARK^®^, Richard Wolf GmbH, Knittlingen, Germany) and a mechanical tissue morcellator (PIRANHA^®^, Richard Wolf, Knittlingen, Germany). The endoscope was connected to a straight fixed camera with a high-definition video system and a flat monitor (KARL STORZ GmbH & Co. KG., Tuttlingen, Germany). We employed a Holmium-YAG laser (Sphinx 100 W, LISA laser products OHG, Kattlenburg-Lindau, Germany) with a 520-µm reusable laser fiber operating at maximum 3.0 J energy,, a 28 Hz, frequency, and a 750 µs pulse duration. These laser parameters were applied consistently across all procedures to ensure uniformity. Normal saline was used for irrigation in all cases. For coagulation of the prostatic fossa, a standard monopolar resectoscope device equipped with a cutting loop or a “roller” probe (SHARK^®^, Richard Wolf, Knittlingen, Germany) was employed.

Data collection was conducted at multiple stages: preoperatively, perioperatively, and during follow-up. Preoperative parameters included patient age, prior prostate resections, prostate biopsies, medication therapies related to prostate enlargement, preoperative antibiotic administration, anticoagulation status, prostate size, PSA levels, presence of preoperative catheters, and bacteria in urine. We also documented the Charlson comorbidity index (CCI), and functional parameters and questionnaires such as the International Prostate Symptom Score (IPSS) and Quality of Life (QoL), International Index of Erectile Function (IIEF), International Consultation on Incontinence Questionnaire (ICIQ), postvoid residual volume (PVR), and maximum uroflow the day before surgery. Perioperative parameters included operative time, resected tissue weight, duration of hospital stay, duration of postoperative catheterization, histopathological results, and postoperative urine culture.

Follow-up evaluations were conducted for all patients between July 2019 and March 2021 in the Department of Urology. During these evaluations, we reassessed functional parameters such as PVR, maximum uroflow, and retrograde ejaculation. Macrohematuria was defined as visible blood in the urine after hospital discharge. Prolonged catheterization was classified as a catheter duration exceeding two postoperative days or the need for re-catheterization. Incontinence was considered present if the patient required more than one pad per day. We also collected responses to the following questionnaires: Freiburg Index of Patient Satisfaction (FIPS) [9], IPSS and QoL, IIEF, and ICIQ. Any complications occurring from the hospital stay up to the follow-up period were also documented. Functional measurements were taken by a urology resident, and a doctoral student undertook the documentation. Questionnaires were distributed during follow-up by the doctoral student, who also documented any interim complications. These follow-up assessment aimed to evaluate the long-term efficacy and safety of 3-HSI technique compared to the three-lobe method.

All parameters were evaluated using the Wilcoxon rank-sum test for baseline comparisons of probe weights and for all non-parametric endpoints, and Fisher’s exact test for binary parameters. Statistical analyses were conducted using IBM SPSS Statistics (Version 23.0 for Windows, IBM Corp., Armonk, NY, USA). Statistical significance was set at *p* < 0.05.

This study was conducted following the principles of the Declaration of Helsinki. Ethics approval was obtained from our institutional review board before initiating the study with the approval reference number 258/18. All participants provided written informed consent before enrollment.

## Results

In our study, a comprehensive comparison of preoperative parameters between patients undergoing the 3-HSI technique (Supplement 1) and those undergoing the classical three-lobe technique revealed several key findings. Notably, there were no significant differences between the two groups regarding patient age (68 ± 7.3 vs. 71.2 ± 7.6, *p* = 0.13), CCI (3.4 ± 1.9 vs. 3.5 ± 1.52, *p* = 0.56), catheterization (25.9% vs. 29.6%, *p* = 1), anticoagulation (11.1% vs. 11.1%, *p* = 1), history of prior prostate surgeries (3.7% vs. 5.5%, *p* = 1) or biopsies (25.9% vs. 16.6%, *p* = 0.38), preoperative antibiotic therapies (37% vs. 48.1%, *p* = 0.47), PSA levels (6.15 ± 6.5 vs. 4.7 ± 4.6, *p* = 0.32), and prostate volume (88 ± 56 vs. 79 ± 49, *p* = 0.66). Furthermore, the baseline assessments from questionnaires such as the IIEF (10.29 ± 8.5 vs. 11.9 ± 8.36, *p* = 0.53) and ICIQ (0.96 ± 2.3 vs. 2.18 ± 3.89, *p* = 0.1), as well as objective measures including maximum uroflow (10.9 ± 4.1 vs. 10.7 ± 7.1, *p* = 0.18) and PVR (97.3 ± 102 vs. 121 ± 310, *p* = 0.28), were similar between groups, indicating similar baseline functional statuses and comorbid conditions (Table 1).

We noted significant differences in the use of prostate-specific medication and severity of lower urinary tract symptoms as measured by the IPSS. Specifically, 55.5% of patients in the 3-HSI group were on prostate-specific medication before surgery, compared to only 11.1% in the three-lobe group (*p* < 0.01). The IPSS was also significantly higher in the 3-HSI group, suggesting a more serious symptom burden in these patients (13.14 ± 6.9 vs. 18.7 ± 6.6, *p* < 0.01) (Table 1).

**Table 1:** Baseline characteristics of the conventionally operated 3-lobe group compared to the group treated with the en-bloc three horse-shoe technique

Baseline characteristics	3-lobe (*n* = 27)	HSI (*n* = 54)	*p*
**Age (y)**	68 ± 7.3 (53–81)	71.2 ± 7.6 (54–83)	0.13
**CCI**	3.4 ± 1.9 (1–10)	3.5 ± 1.52 (1–8)	0.56
**Previous prostate surgery**	1/27 (3.7%)	3/54 (5.5%)	1
**Previous prostate biopsy**	7/27 (25,9%)	9/54 (16,6%)	0.38
**Medication therapy**	3/27 (11.1%)	30/54 (55.5%)	< 0.01
**Presence of urinary catheter**	7/27 (25.9%)	16/54 (29.6%)	1
**Anticoagulation**	3/27 (11.1%)	6/54 (11.1%)	1
**Positive urinary culture** **preoperatively**	0/27 (0%)	3/54 (5.5%)	0.54
**Antibiotic therapy**	10/27 (37%)	26/54 (48.1%)	0.47
**PSA (ng/ml)**	6.15 ± 6.5 (0.7–31.7)	4.7 ± 4.6 (0.74-26)	0.32
**Prostate volume (g)**	88 ± 56 (20–250)	79 ± 49 (30–280)	0.66
**IPSS**	13.14 ± 6.9 (1–32)	18.7 ± 6.6 (7–34)	< 0.01
**QoL**	3.1 ± 1.08 (1–5)	3.37 ± 1.08 (0–5)	0.18
**IIEF-5**	10.29 ± 8.5 (0–25)	11.9 ± 8.36 (0–25)	0.53
**ICIQ**	0.96 ± 2.3 (0–9)	2.18 ± 3.89 (0–18)	0.1
**Max. Uroflow (ml/s)**	10.9 ± 4.1 (2.2–19.5)	10.7 ± 7.12 (3–38)	0.18
**PVR (ml)**	97.3 ± 102 (0-350)	121 ± 310 (0-2200)	0.28

CCI = Charleston Comorbidity Index. IPSS = International Prostate Symptom Score. QoL = Quality of Life. IIEF-5 = International Index of Erectile Function. ICIQ = International Consultation on Incontinence Questionnaire. PVR = Postvoid Residual Volumen

We made several key observations when comparing the perioperative parameters of the 3-HSI and classical three-lobe techniques. There were no significant group differences in terms of the size of the resected prostate tissue (71 ± 65 vs. 55 ± 41, *p* = 0.68), duration of hospitalization (3.5 ± 1.3 vs. 3.9 ± 1.86, *p* = 0.2), length of postoperative catheterization (2.1 ± 0.53 vs. 2.56 ± 1.58, *p* = 0.19), or proportion of histopathologically malignant results (7.4% vs. 7.4%, *p* = 1). These findings suggest that both surgical techniques are similar in these key perioperative outcomes, indicating similar levels of efficacy in tissue resection and immediate postoperative recovery. However, a significant difference became apparent in the operative time, with the 3-HSI technique demonstrating a clear advantage. The 3-HSI group’s mean operative time was notably shorter than the three-lobe group’s (60.1 ± 34 vs. 44.9 ± 43, *p* = 0.01) (Table 2). To mention, a hospitalization period of four days is standard at our institution; however, in cases of an uncomplicated postoperative course and ASA I–II status, discharge may occur as early as postoperative day three.

**Table 2:** Comparison of perioperative parameters between the group treated with the conventional 3-lobe technique and the group undergoing the en-bloc three horse-shoe approach

Perioperative Parameters	3-lobe (*n* = 27)	HSI (*n* = 54)	*p*
**Positive postoperative urinary culture**	0/27 (0%)	3/54 (5.5%)	0.54
**Duration of Operation [min]**	60.1 ± 34 (15–140)	44.9 ± 43 (14–280)	0.01
**Duration of catheterization [d]**	2.1 ± 0.53 (1–4)	2.56 ± 1.58 (2–11)	0.19
**Resectat [g]**	71 ± 65 (10–235)	55 ± 41 (14–210)	0.68
**Hospitalization [d]**	3.5 ± 1.3 (3–9)	3.9 ± 1.86 (2–13)	0.2
**Malignant pathology**	2/27 (7.4%)	4/54 (7.4%)	1

Our follow-up data revealed that the three-lobe group were assessedafter a significantly longer period compared to the 3-HSI group (1161 ± 320 vs. 779 ± 300, *p* < 0.01). There were no significant differences between groups during the follow-up period in terms of complications classified as Clavien-Dindo grade 1 and 2. Grade 1 complications included macrohematuria (37% vs. 33.3%, *p* = 0.8), incontinence (29.6% vs. 40.7%, *p* = 0.46), prolonged catheterization (18.5% vs. 22.2%, *p* = 0.77), urinary retention requiring recatheterization (3.7% vs. 11.1%, *p* = 0.41), and dysuria (22.2% vs. 9.2%, *p* = 0.16). Grade 2 complications encompassed urinary tract infections (11.1% vs. 5.5%, *p* = 0.39), transfusions (0% vs. 1.8%, *p* = 1), and epididymitis (3.7% vs. 0%, *p* = 0.33) (Table 3).

Regarding Clavien-Dindo grade 3 complications, no significant differences were observed in grade 3a complications, specifically clot retention, between groups (0% vs. 1.8%, *p* = 1). Similarly, concerning grade 3b complications, there were no significant differences in postoperative bleeding requiring surgical intervention (0% vs. 3.7%, *p* = 0.55) or recurrent adenomas (0% vs. 3.7%, *p* = 0.55). Urethral strictures were less frequent in the 3-HSI group than in the three-lobe group without statistical significance (7.4% vs. 1.8%, *p* = 0.25)(Table 3).

Functional parameters including maximum uroflow (25.6 ± 17 vs. 22.7 ± 14.8, *p* = 0.39) and PVR (51.2 ± 38.8 vs. 58.5 ± 126, *p* = 0.22), as well as responses to questionnaires such as the IPSS (5.6 ± 4.28 vs. 7.12 ± 6.77, *p* = 0.69), QoL (1.1 ± 0.95 vs. 1.25 ± 1.1, *p* = 0.77), IIEF (8.7 ± 8.3 vs. 11.03 ± 8.59, *p* = 0.26), IICIQ (1.1 ± 2.4 vs. 2.2 ± 3.1, *p* = 0.13), and FIPS (1.7 ± 0.56 vs. 1.9 ± 0.89, *p* = 0.18), showed no significant differences between groups. These results suggest that both techniques yield similar long-term functional outcomes and patient-reported satisfaction (Table 3).

**Table 3:** Comparison of postoperative parameters between the conventionally operated group using the 3-lobe technique by gilling (3-lobe) and the three horse-shoe incision group at follow-up examination

	3-lobe (*n* = 27)	HSI (*n* = 54)	*p*
**Duration to Follow_Up [d]**	1161 ± 320 (728–1861)	779 ± 300 (454–1623)	< 0.01
**Clavien-Dindo 1**			
**Macrohematuria**	10/27 (37%)	18/54 (33.3%)	0.8
**Incontinence**	8/27 (29.6%)	22/54 ( 40.7%)	0.46
**Prolonged Catheterization**	5/27 (18.5%)	12/54 (22.2%)	0.77
**Urinary Retention**	1/27 (3.7%)	6/54 (11.1%)	0.41
**Dysuria**	6/27 (22.2%)	5/54 (9.2%)	0.16
**Clavien-Dindo 2**			
**Urinary infection**	3/27 (11.1%)	3/54 (5.5%)	0.39
**Transfusion**	0/27 (0%)	1/54 (1.8%)	1
**Epididymitis**	1/27 (3.7%)	0/54 (0%)	0.33
**Clavien-Dindo 3**			
**Clot Retention**	0/27 (0%)	1/54 (1.8%)	1
**Bleeding**	0/27 (0%)	2/54 (3.7%)	0.55
**Stricture**	2/27 (7.4%)	1/54 (1.8%)	0.25
**Recurrent Adenoma**	0/27 (0%)	2/54 (3.7%)	0.55
**FIPS**	1.7 ± 0.56 (1–3)	1.9 ± 0.89 (1–5)	0.18
**IPSS**	5.6 ± 4.28 (0–18)	7.12 ± 6.77 (0–27)	0.69
**QoL**	1.1 ± 0.95 (0–4)	1.25 ± 1.1 (0–4)	0.77
**IIEF-5**	8.7 ± 8.3 (0–25)	11.03 ± 8.59 (0–25)	0.26
**ICIQ**	1.1 ± 2.4 (0–9)	2.2 ± 3.1 (0–13)	0.13
**Max. Uroflow [ml/s]**	25.6 ± 17 (4.5–78)	22.7 ± 14.8 (7-70.2)	0.39
**PVR [ml]**	51.2 ± 38.8 (0-152)	58.5 ± 126 (0-910)	0.22
**Retrograde Ejaculation**	21/27 (77.7%)	44/54 (81,4%)	0.77

FIPS = Freiburg Index of Patient Satisfactory. IPSS = International Prostate Symptom Score. QoL = Quality of Life. IIEF-5 = International Index of Erectile Function. ICIQ = International Consultation on Incontinence Questionnaire. PVR = Postvoid Residual Volumen

## Discussion

HoLEP has emerged as a superior technique for alleviating bladder outlet obstruction (BOO)-related LUTS, particularly in patients with larger prostate glands [10]. Despite its advantages, namely better functional outcomes and less bleeding, HoLEP is often associated with a steep learning curve, which can be a barrier to widespread adoption [11]. Miernik & Schöb (2019) hypothesized that eliminating the need for longitudinal incisions and preserving anatomical landmarks could lead to greater standardization and potentially lower the learning curve [7]. In this study, we compared the novel “Three Horseshoe Incision” (3-HSI) HoLEP technique with the traditional three-lobe method, utilizing an adequate follow-up period to assess results.

Regarding perioperative parameters, the 3-HSI group demonstrated a shorter operative time, supporting the hypothesis that the en-bloc technique enables more efficient preparation. This finding suggests that it is advantageous not to remove each of the prostate’s three lobes individually, as the traditional three-lobe method requires. The 3-HSI technique’s streamlined process likely contributes to shortening the operative time. Several en-bloc techniques have been published, each aiming to optimize operative times and shorten the learning curve. Scoffone and Cracco (2016) describe an en-bloc no-touch technique that minimizes energy delivery to the capsule [12]. Another approach is the MOSES technique involving early apical release, both of which demonstrate the optimization potential of en-bloc techniques regarding operative time [13]. Recent studies further support the efficiency of en-bloc HoLEP over the traditional three-lobe technique. A propensity score-matched analysis of 606 patients found that en-bloc enucleation resulted in shorter enucleation times and required less than half the total laser energy compared to the three-lobe approach [14]. These findings align with our results, supporting the shorter operative time observed with en-bloc techniques, including 3-HSI.

Our study’s postoperative outcomes were also similar between groups. It is noteworthy that during follow-up, the HSI group’s prostate-specific symptom burden, despite being more pronounced preoperatively, resembled the control group’s, indicating sufficiently alleviated symptoms. Several studies have demonstrated that en-bloc HoLEP techniques provide similar safety profiles to the traditional three-lobe method, with no significant differences in postoperative complications. Our findings are consistent with previous research showing comparable rates of macrohematuria, urinary retention, and urinary tract infections, further supporting the safety of the 3-HSI technique [4, 15]. Additionally, the incidence of urethral strictures and recurrent adenomas in our study was low and aligns with previous reports on HoLEP techniques. A study by Elkoushy et al. reported a 10-year reoperation-free probability of 95.1% following HoLEP, indicating a low rate of recurrent adenomas [16]. Although our three-lobe group showed a tendency toward a higher stricture rate, this difference was not statistically significant due to the smaller sample size and is therefore not fully comparable to larger database studies. Regarding functional outcomes, our results confirm that both techniques lead to significant improvements in IPSS, uroflow parameters, and postvoid residual volume (PVR), with no notable differences between groups. These findings align with previous studies demonstrating that en-bloc HoLEP achieves comparable long-term symptom relief and bladder emptying efficiency compared to the three-lobe approach [17].

Many newly developed techniques aim to minimize incontinence following surgical treatment. However, we observed no advantage in this regard in our study. Several studies have described techniques reducing postoperative incontinence. Elshal et al. (2023) introduced the veil-sparing HoLEP (VS-HoLEP) technique, which significantly reduced early incontinence rates compared to standard HoLEP [18]. Shishido et al. (2023) found that anteroposterior dissection HoLEP led to a lower incidence of stress urinary incontinence and faster recovery than traditional methods [6]. Zhou et al. (2022) showed that an en bloc technique involving urethral mucosal flap sparing reduced both short-term and long-term incontinence more than conventional HoLEP [15]. These techniques highlight ways to improve continence outcomes. Interestingly, in a direct comparison study, our technique demonstrated the lowest incontinence rate at three months compared to the original en-bloc method and a technique involving early mucosal strip detachment. However, it is important to note that the continence rates of all techniques converged by six and 12 months; the technique has since been modified to include early apical release, which complicates a straightforward comparison [19].

This study has limitations to be considered when interpreting our results. Firstly, it is a monocentric study conducted by a single surgeon, which could impact the generalizability of our findings. Secondly, the group sizes were unequal, as our participants had to be recruited from the existing patient pool. To form comparable groups, we selected only those patients willing to undergo follow-up. This limitation might introduce some bias in the results, as the characteristics of patients who were willing to participate in follow-up might differ from those who were not. Thirdly, in the three-lobe group, only the total operative time was initially recorded, without separately documenting enucleation and morcellation times, making a direct comparison of enucleation efficiency between the techniques impossible. Additionally, the longer mean follow-up duration in the three-lobe group could introduce bias when comparing functional and complication outcomes. Furthermore, the retrospective, non-randomized study design without propensity score matching limits the control of potential confounding variables.

## Conclusion

The 3-HSI HoLEP technique appears to be a safe and effective alternative to the traditional three-lobe approach, with comparable long-term outcomes and patient satisfaction. Notably, it offers improved operative efficiency through shorter operative times. These findings support the consideration of 3-HSI as a viable option within the spectrum of established HoLEP techniques.

## Supplementary Information

Below is the link to the electronic supplementary material.

Supplementary Material 1

## Data Availability

The datasets used and analysed during the current study are available from the corresponding author on reasonable request.

## Abbreviations

BOO: Bladder Outlet Obstruction
BPH: Benign Prostatic Hyperplasia
CCI: Charlson Comorbidity Index
FIPS: Freiburg Index of Patient Satisfaction
HoLEP: Holmium Laser Enucleation of the Prostate
ICIQ: International Consultation on Incontinence Questionnaire
IIEF: International Index of Erectile Function
IPSS: International Prostate Symptom Score
LUTS: Lower Urinary Tract Symptoms
PVR: Postvoid Residual Volume
QoL: Quality of Life
TUR-P: Transurethral Resection of the Prostate
3-HSI: Three horse shoe-like incision
