# Supplementary material for: Long-term follow-up of “three horse shoe-like incision (3-HSI) holmium laser enucleation of the prostate: outcomes and efficacy of a novel en-bloc technique for anatomic transurethral prostatectomy”
Source: BMC Urol. 2025 Jul 31;25:185. doi: 10.1186/s12894-025-01885-6 (PMC12312310; doi:10.1186/s12894-025-01885-6)
Supplement: Supplementary file 1 — Supplementary Material 1 [file 12894_2025_1885_MOESM1_ESM.docx]

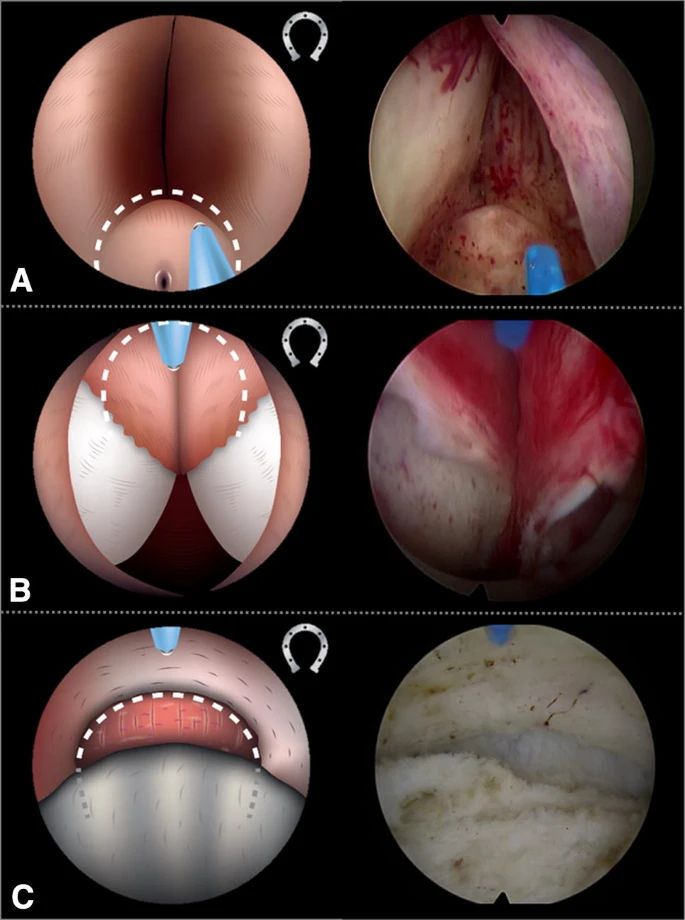


*Supplement 1.* Schematic overview of the key surgical steps of the 3-Horse Shoe Incision (3-HSI) technique. *(A) Paracollicular incision, (B) Mucosal strip dissection, (C) Bladder neck incision.* This figure is adapted from the original publication by Miernik & Schöb [7] and is included in this paper with their permission.
